# Nephroprotective impact of royal jelly against rhabdomyolysis-induced kidney damage in rats

**DOI:** 10.22038/ijbms.2025.79750.17275

**Published:** 2025

**Authors:** Ahmad Reza Aminian, Mohammad Taghi Khoshniat, Abolfazl Khajavi Rad, Maryam Mahmoudabady, Mohammad Hossein Rigi, Sara Hosseinian

**Affiliations:** 1 Department of Physiology, School of Medicine, Mashhad University of Medical Sciences, Mashhad, Iran; 2 Applied Biomedical Research Center, Mashhad University of Medical Sciences, Mashhad, Iran

**Keywords:** Acute kidney injury, Apoptosis, Inflammation, Oxidative stress, Rhabdomyolysis, Royal jelly

## Abstract

**Objective(s):**

The current work aimed at studying the impact of royal jelly on kidney damage caused by rhabdomyolysis in male rats.

**Materials and Methods:**

40 male rats were randomly assigned to five groups of eight: control, rhabdomyolysis, and rhabdomyolysis, administered with three doses of royal jelly (RJ) (100, 200, and 400 mg/kg) for seven days. On the fifth day, we injected glycerol intramuscularly to induce rhabdomyolysis. Researchers examined serum biochemical parameters, inflammation, oxidative stress, apoptosis, and kidney tissue injury.

**Results:**

As a result of injecting glycerol, serum levels of creatinine, urea, and creatine phosphokinase were considerably elevated. The concentration of inflammatory mediators, as well as the expression of apoptotic parameters, was significantly elevated after glycerol injection. The percentage of kidney tissue damage and neutrophil gelatinase-associated lipocalin expression also increased significantly. Oral usage of RJ (100, 200, and 400 mg/kg) caused a decline in serum CPK, tissue level of total thiols, catalase activity, and renal expression of *BAX* compared to the rhabdomyolysis group. Serum creatinine and NGAL expression were also significantly reduced by the RJ (200 and 400 mg/kg). RJ significantly reduced the concentration of inflammatory mediators at 100 and 200 mg/kg doses and the expression of *bcl2* by RJ at 100 and 400 mg/kg doses.

**Conclusion:**

Royal jelly safeguards the kidney from rhabdomyolysis-related damage, primarily through its anti-oxidant, anti-apoptotic, and anti-inflammatory effects.

## Introduction

**Figure 1:**
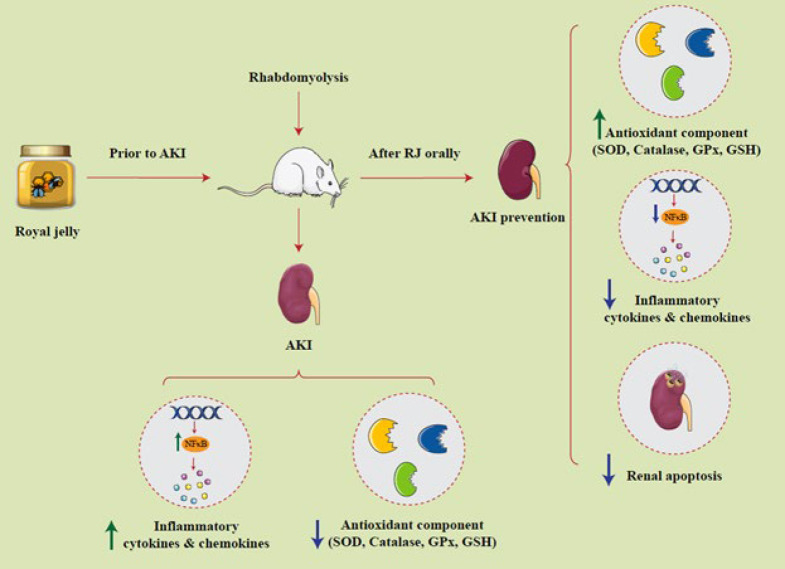
The effect of Royal jelly against kidney injury induced by rhabdomyolysis in rat

Rhabdomyolysis is described by muscle fibers’ necrosis and rapid striated muscle tearing (1). As a result of this process, the breakdown products of the cells are released into the bloodstream and the extracellular space (2). In severe cases, severe electrolyte imbalances and acute kidney failure (AKI) are life-threatening (3). Heme proteins released from muscle after rhabdomyolysis are metabolized in the kidneys, generate free radicals, collect nitric oxide (NO), and trigger endothelin receptors (4). These factors work together to enhance renal vasoconstriction, leading to intraluminal obstruction and, ultimately, myoglobinuric acute renal failure (5). After muscle damage or ATP depletion, excess Na+ and Ca^2+^ flow into the cytoplasm (5). An elevation in intracellular Na+ pulls water into the cell, compromising the integrity of the intracellular environment (6). Extended elevation of intracellular Ca^2+^ levels also results in sustained contraction and further depletion of ATP (6). These alterations in the muscle cell environment result in an inflammatory and self-sustaining myolytic cascade that leads to muscle fiber necrosis and releases muscle components into the bloodstream and extracellular space (7). A rhabdomyolysis consequence is acute kidney injury (AKI) that occurs in 10 to 40 percent of cases (8). Increased urinary excretion of myoglobin, or myoglobinuria, is a significant factor in AKI pathophysiology developed by rhabdomyolysis (9). Within the tubular cell, the iron in myoglobin is transformed into ferrous iron, generating hydroxyl radicals (10). During rhabdomyolysis, circulating blood volume is reduced for extracellular fluid outflow into the injured tissue. Also, renin-angiotensin system activation and renal vasoconstriction result in reduced renal blood flow and, consequently, disturbances in renal small blood flow, which leads to hypoxia (11). In rhabdomyolysis, recent reports have identified new markers for early prediction of AKI in urine and blood (12). Because these biomarkers show the emergence of early mechanisms before the loss of renal function, they enable an earlier diagnosis (12). The most extensively studied biomarker for rhabdomyolysis is neutrophil gelatinase-associated lipocalin (13, 14). Its production and release rise quickly in response to toxic or ischemic injury, making it noticeable in blood and urine as a specific and sensitive biomarker for renal injury (15). Using compounds with anti-inflammatory and anti-oxidant effects could be an appropriate approach for reducing the damage caused by rhabdomyolysis (16). Royal jelly (RJ) is a mixture of secretions from the mandibular glands of honey bees, encompassing a diverse array of chemical compounds, including proteins (dry weight 17–45%), royalism (with strong antibacterial properties), jellies (with antimicrobial action), carbohydrates, fatty acids, vitamins (thiamin, B vitamins, pentatonic acid, riboflavin, niacin, biotin, and folic acid) (17). The anti-oxidant action of RJ prevents lipid peroxidation and oxidative stress, protecting DNA from oxidative stress (Figure 1) (18). Kanbur *et al*. (2009) stated that parenterally fed rats presented higher anti-oxidant concentrations in the liver and serum and a longer average lifespan (19). In addition, Karadeniz *et al*. (2011) showed that maternal intake reduces oxidative stress and cisplatin-induced nephrotoxicity (20). This work investigates RJ’s antiapoptotic, anti-oxidant, and anti-inflammatory capacity on AKI caused by rhabdomyolysis in rats.

## Materials and Methods

We used forty male Wistar rats (250–300 grams) from the Medical Faculty animal house of Mashhad University of Medical Sciences to conduct this experimental study. Rats stayed at a temperature of 22 ± 2 °C with free access to water and food in a 12-hr dark and 12-hr light cycle. Ethical cases of working with experimental animals during the research were employed in line with procedures sanctioned by the Faculty Ethics Committee. The animals were placed randomly into five groups of eight, as detailed below:

### Control

The animals were given normal saline orally for one week and injected intramuscularly on the fifth day.

### Rhabdomyolysis

In this group, the animals were administered normal saline orally for seven days. On the fifth day, they were first anesthetized with 70 mg/kg ketamine and 7 mg/kg xylazine, after which 50% glycerol (10 ml/kg) was administered intramuscularly in the hind leg muscles (21).

### Rhabdomyolysis + RJ (100 mg/kg)

The animals were administered RJ (100 mg/kg) via gavage for seven days, and on the research’s fifth day, glycerol was injected intramuscularly, like the second group (22).

### Rhabdomyolysis + RJ (200 mg/kg)

The samples were administered RJ (200 mg/kg) via gavage for seven days, and they underwent glycerol intramuscular injection on the fifth day, similar to the second group (23).

### Rhabdomyolysis + RJ (400 mg/kg)

The animals were administered RJ (400 mg/kg) via gavage for seven days, and like the second group (20), they received glycerol intramuscular injection on the fifth day.

On the study’s final day (day 7), after weighing the rats, serum specimen was gathered. The kidneys were then swiftly excised and weighed up before the rats were humanely euthanized. Urea, CPK, creatinine, calcium, and potassium levels in serum were evaluated using Pars Azmoon kits (Iran). The right kidney was fixed in 10% formalin for histological assessment, while the left kidney was kept at −80 °C for evaluations of apoptosis, inflammation, and oxidative stress.

### Assessment of rhabdomyolysis induction and kidney function

Creatinine, calcium, CPK, and urea serum levels were determined using commercial kits. Serum Na concentrations were analyzed using a quantitative electrode quantification technique (Electrolyte Analyzer, AC 980, China).

### Examination of kidney injury biomarker and apoptosis in kidney tissue

*RNA extraction and cDNA synthesis*

Total RNA extraction was done from homogenized kidney tissue using the Favorgen RNA extraction kit (Favorgen Biotech) in line with the producer’s procedures. We used NanoDrop to check the purity and quality of the separated RNA (Thermo 2000, USA). 500 ng of total RNA was employed to generate cDNA using a cDNA synthesis kit (YektaTajhiz Azma cDNA Synthesis Kit, Iran) and a BioRad Thermal Cycler (Bio-Rad Laboratories, USA) according to protocol instructions. 

*Real-time quantitative polymerase chain reaction*

We employed Real Q Plus 2x Master Mix Green for performing quantitative real-time polymerase chain reaction in these conditions: 15 min at 95 °C, followed by 45 cycles of 30 sec at 95 °C and 60 sec at 60 °C, with default melting settings. The total volume of the reaction mix was 25 μl, consisting of forward primer (0.5 μl), diluted cDNA (2 μl), reverse primer (0.5 μl), and Master Mix Green (12.5 μl), topped up to 25 μl with nuclease-free water. The messenger RNA (mRNA) expression levels of target genes were normalized to GAPDH, a housekeeping gene, and compared to a control gene. The Livak approach (2-∆∆CT) was utilized to evaluate the relative gene expression (24). Table 1 presents the primers used and developed with primer3 software based on gene sequences obtained from NCBI Gene. Their specificity was verified using NCBI Blast.

*Analysis of histopathology*

Following fixing in 10% formalin, researchers dehydrated the right kidneys in graded alcohols, followed by embedding in paraffin. Tissue sections of 5 µm were created, and the slides were stained using the hematoxylin-eosin approach for microscopic analysis. Samples were evaluated by a scoring system from 0 to 4, where 0= no tissue injury, 1= 1–25% destruction of tissue, 2= 25–50% destruction of tissue, 3= 50–75% destruction of tissue, and 4= 75–100% destruction of tissue. 

*Evaluation of anti-oxidant enzyme and oxidative stress markers*

MDA, an indicator of oxidative stress, creates a red-colored complex when it reacts with TBA, referred to as TBARS, achieving peak absorbance at 535 nm. To prepare the mixture, TCA (15 g), HCl (2 ml), and TBA (0.375 g) were mixed, and then 2 ml of the mix was combined with 1 ml of serum or kidney homogenate in a centrifuge tube, being warmed in a water bath for 50 min. After being cooled, the combination was centrifuged at 1000 rpm for 10 min. The absorbance (A) of the resulting layer was measured at 535 nm (25). The MDA concentration was found using this relation: C (M) = A/1.56 ×105.

The total thiol content in the renal tissue was determined by the equation provided by Sedlak and Lindsay (1968) (26). A volume of 50 μl of supernatant was combined with Tris-EDTA buffer (1 ml), and absorbance was measured against a Tris-EDTA buffer alone at 412 nm (A1). Afterward, 20 μl of the DTNB reagent (10 mM in methanol) was combined, and the absorbance was reevaluated after 10 min (A2). The DTNB reagent’s absorbance was recorded as a blank (B). The total thiol concentration (mM) was obtained using this relation:

Total thiol concentration (mM) = (A2-A1-B) × 1.07/0.05 × 13.6

Aebi’s calorimetric method, which is based on the reduction of H_2_O_2_, was employed for measuring catalase action in kidney tissue homogenates (27). 

*Evaluation of inflammation in kidney tissue*

The IL-1β and TNF-α concentrations in the left kidneys were measured using an ELISA assay, following the manufacturer’s instructions (Karmania Pars Gene Co, Iran).

### Statistical analysis

Research data were presented as means ± SEM. ANOVA was used to evaluate the difference between means, followed by the Tukey test. Differences were statistically significant at *P*<0.05.

## Results

### RJ impact on serum biochemical parameters

On the research’s final day (48 hr after injecting glycerol), the serum level of CPK presented a noticeable elevation in comparison to the control group (*P*<0.001). On this day, serum urea and creatinine concentrations also elevated considerably in comparison with the control group (*P*< 0.01- *P*<0.001) (Table 2). Serum calcium concentration did not illustrate any significant alteration between different experimental groups. In RJ-treated groups at 100, 200, and 400 mg/kg doses, serum CPK decreased compared to the Rhabdo group (*P*<0.001 for all). In the Rhabdo+RJ400 group, creatinine and serum urea concentration showed a significant reduction in comparison with those of the Rhabdo group (*P*<0.001 for all) (Table 2). Also, serum creatinine concentration was significantly reduced in the Rhabdo+RJ200 group than in the Rhabdo group (*P*<0.05). The serum level of potassium in the Rhabdo+RJ200 group was significantly elevated compared to the Rhabdo group (*P*<0.01) (Table 2). Serum calcium levels were significantly lower in all groups receiving royal jelly than in the Rhabdo group (*P*<0.001 for all). In comparison with the Rhabdo+RJ100 group, the concentration of creatinine and serum urea was reduced (*P*<0.01 for both) (Table 2).

### RJ impact on expression of apoptotic mediators and kidney injury marker

**Figure 2:**
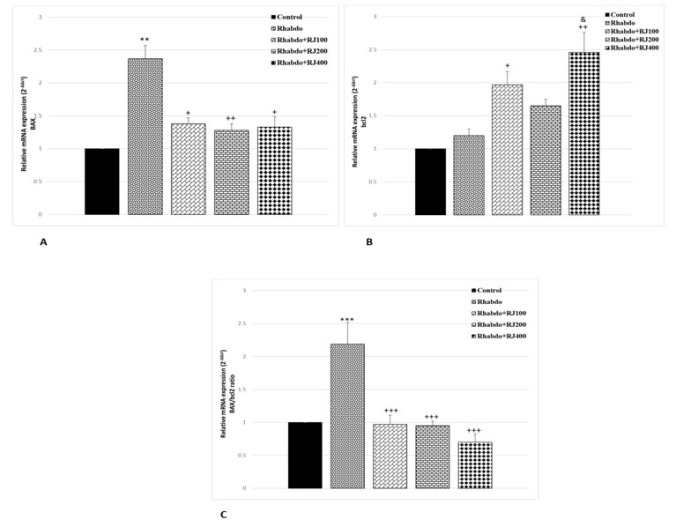
Renal expression of *BAX* (A), *bcl2* (B), and BAX/bcl2 ratio (C) among different experimental groups of rats

BAX gene expression in the Rhabdo group presented a significant rise compared with the control group (*P*<0.01). Meanwhile, gene expression in all treated RJ groups presented a significant decline in comparison with the Rhabdo group (*P*<0.05-*P*<0.01) (Figure 2A). Conversely, bcl2 gene expression showed no significant change in the Rhabdo group in comparison with control rats. Nevertheless, in the RJ-treated groups at 100 and 400 mg/kg doses, bcl2 gene expression was significantly elevated compared to the Rhabdo group (*P*<0.05–*P*<0.01) (Fi). Consequently, the apoptosis index (Bax/Bcl-2 ratio) increased in the Rhabdo group in comparison to the control group (*P*<0.001). On the other hand, the Bax/Bcl-2 ratio was significantly reduced in all RJ-treated groups in comparison with those of the Rhabdo group (*P*<0.001) (Figure 2C). 

**Figure 3:**
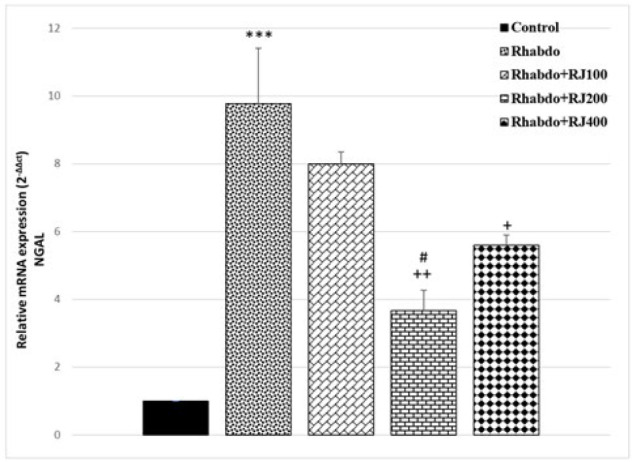
Renal expression of NGAL in different experimental groups of rats

Injection of glycerol resulted in a notable rise in NGAL expression as a kidney injury marker when compared to the control group (*P*<0.001) (Figure 3). Nevertheless, we noted a considerable decline in NGAL expression in Rhabdo+RJ200 and Rhabdo+RJ400 in comparison to the Rhabdo group (*P*<0.05-*P*<0.01) (Figure 3). 

### RJ’s impact on kidney inflammation

**Figure 4:**
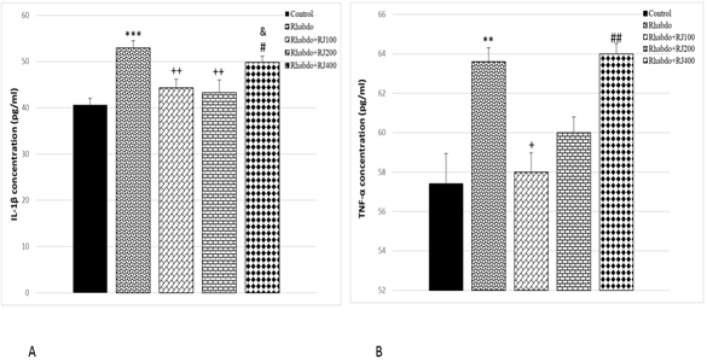
Renal tissue concentrations of IL-1β (A) and TNF-α (B) in in different experimental groups of rats

Kidney TNF-α and IL-1β protein levels showed a significant elevation in response to glycerol injection compared to control animals (*P*<0.01–*P*<0.001). RJ administration at 100 and 200 mg/kg doses during rhabdomyolysis significantly decreased renal production of IL-1β compared to the Rhabdo group (*P*<0.001). Nevertheless, treating the animals with RJ400 could not reduce kidney IL-1β concentration significantly. TNF-α concentration in the Rhabdo+RJ100 group significantly declined compared to the Rhabdo group (*P*<0.05). Nevertheless, treating rats with RJ 200 and 400 could not reduce kidney TNF-α concentration significantly (Figure 4).

### Impact of RJ on kidney histopathology

**Figure 5:**
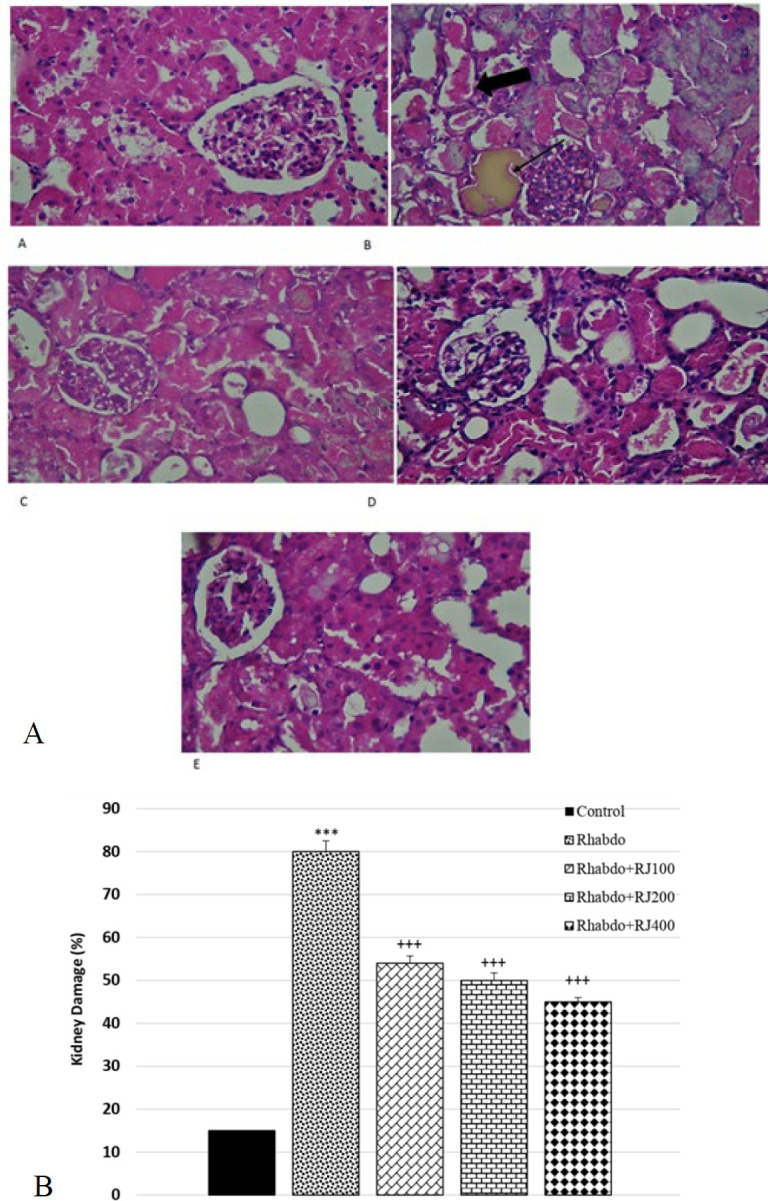
Light microscopy of renal section (A) and the percentage of kidney tissue damage (B) in different experimental groups of rats

Kidney tissue sections in the control group exhibited a typical structure (Figure 5A). Glycerol injection caused tubular epithelial degeneration and necrosis, marked intratubular hemoglobin, and hyaline cast formation, consistent with hemorrhage and vacuolization in epithelial cells. Compared with the Rhabdo group, RJ treatment improved kidney histological abnormalities (Figure 5A). Kidney damage in the Rhabdo group was significantly increased compared to the control group (*P*<0.001). RJ therapy at doses of 100, 200, and 400 mg/kg significantly improved histopathology alteration compared to the Rhabdo group (*P*<0.001 for all) (Figure 5B).

### RJ impact on oxidant/anti-oxidant balance in the kidney

**Figure 6:**
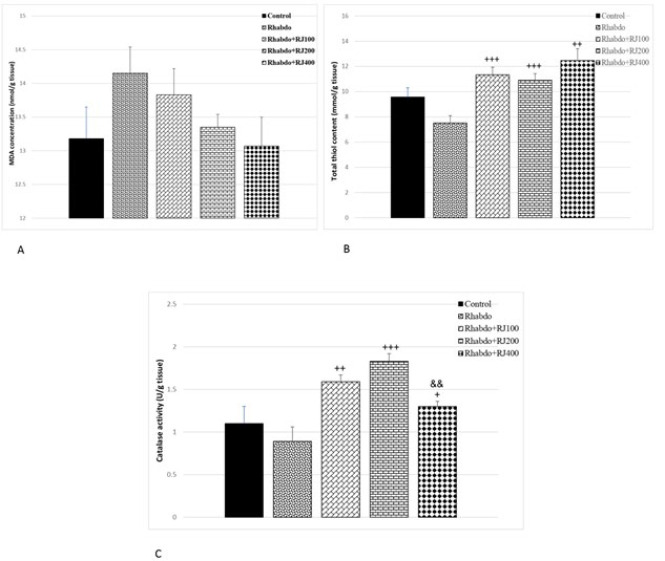
Renal tissue concentrations of MDA (A), total thiols (B), and catalase activity (C) in all experimental groups of rats

MDA concentration in the kidney tissues of rats did not significantly change among different experimental groups (Figure 6A). The total thiol content in the kidney tissues of the Rhabdo group was 22% lower than that of the control group, although this difference was not statistically significant. Administration of RJ for one week in the Rhabdo+RJ100, Rhabdo+RJ200, and Rhabdo+RJ400 groups presented a significant rise in total thiol content in comparison to the Rhabdo group (*P*<0.01–* P*<0.001) (Figure 6B). We did not note any significant differences in catalase activity in the Rhabdo group compared to the control. However, animals in all RJ-treated groups had higher kidney catalase enzyme activity compared to the Rhabdo group (*P*<0.05–*P*<0.001) (Figure 6C).

## Discussion

Skeletal muscle damage is the hallmark of rhabdomyolysis, which is typically linked to trauma but can also arise from several clinical situations, such as infection, exposure to toxins and drugs, abrupt temperature changes, intense physical activity, and prolonged muscle compression. One effective laboratory model for inducing rhabdomyolysis is glycerol’s intramuscular injection (28). In this study, a single intramuscular injection of 50% glycerol was administered to induce rhabdomyolysis on the fifth day of the experiment. The increase in serum CPK, caused by the lysis of the muscle cell membrane, is an essential sign of rhabdomyolysis. In this work, a significant elevation was noted in the glycerol-injected group. The most dangerous consequence of rhabdomyolysis is AKI (2). The mechanisms through which rhabdomyolysis results in AKI are renal vasoconstriction, oxidative stress, inflammation, myoglobin nephrotoxicity, and tubular obstruction by myoglobin casts (29). After rhabdomyolysis, fluid enters muscle cells through ruptured membranes, decreasing extracellular fluid volume. Vasopressin is released; the renin-angiotensin and sympathetic nervous systems are triggered due to this extracellular volume depletion (30). These vasoconstrictors decrease renal blood flow, resulting in a decline in glomerular filtration rate and an increase in the preservation of nitrogenous waste products, including creatinine and urea, as seen in our findings (31). Severe myoglobinuria is a primary contributor to oxidative stress following rhabdomyolysis (32). In this work, despite a 20% reduction in total thiol group content, MDA concentration and catalase activity did not show significant changes after induction of rhabdomyolysis. This is because of time-dependent characteristics of oxidative stress in kidney tissue after rhabdomyolysis. However, royal jelly increases catalase enzyme activity and thiol content in the kidney by strengthening the anti-oxidant system. These effects are likely caused by the anti-oxidant effect and free radical scavenging actions of royal jelly (33). Royal jelly appears to reduce oxidative damage by the production of ROS and to reduce NADPH oxidase 2 activity. By NO formation and reducing lipid peroxidation, along with increasing the available GSH capacity and upstream anti-oxidants in the kidney, royal gel showed that it could save kidney tissue in the AKI model (20). It has been shown that activating NF-κB and toll-like receptors (TLRs) increases the production of inflammatory cytokines in the kidney tissue, contributing to the development of AKI following rhabdomyolysis (34). In our work, TNF-α and IL-1β elevated in the glycerol group, and 100 and 200 mg/kg of royal jelly were able to lower the concentration of these inflammatory mediators. These findings came in line with previous studies (35-37). Besides the anti-oxidant effects, various works have shown the anti-inflammatory capacities of RJ, which can justify our findings (38-41). Mitochondria are the main regulators of oxidative stress and cell apoptosis. Following rhabdomyolysis, the construction of ROS induces mitochondrial dysfunction and activates caspases and tubular cell apoptosis (42). In our work, the expression of apoptotic genes and the apoptotic index, the ratio of *BAX*/*bcl2*, increased in the rhabdomyolysis group. Therefore, given the considerable function of oxidative stress in inducing apoptosis, downregulation of *BAX* and upregulation of *bcl2* in the RJ-treated groups can be caused by its anti-oxidant effects. NGAL, as a stress protein, is upregulated in the early phases of AKI, and numerous research works have indicated that NGAL, as a biomarker of renal damage, is quickly released in response to tubular damage (43, 44). The serum NGAL levels in AKI cases are connected to the kidney damage severity and the mortality risk (45). Interestingly, this marker is increased by oxidative stress and inflammation. Therefore, in our study, in line with other studies, NGAL overexpressed following rhabdomyolysis and decreased by RJ at 200 and 400 mg/kg doses. This impact of RJ can be caused by its anti-inflammatory and anti-oxidant capacities (38-40). 

**Table 1:** List of primer sequences of rat BAX, bcl2, and NGAL genes

**Gene**	**Primer sequence 5** **ʹ** **–** **3** **ʹ**	**Total length amplified**
***BAX***	F 5ʹ- CACCTGAGCTGACCTTGGAG-3ʹ	125
	R 5ʹ- CACATCAGCAATCATCCTCTGC-3ʹ	
***BCL2***	F 5ʹ- GATAACGGAGGCTGGGATGC-3ʹ	92
	R 5ʹ- AGCAGCGTCTTCAGAGACAG-3ʹ	
***GAPDH***	F 5ʹ- CTTCTCTTGTGACAAAGTGGACA -3ʹ	117
	R 5ʹ- TTGACTGTGCCGTTGAACTTG -3ʹ	
**NGAL**	TCACCCTGTACGGAAGAACC	84
	CCTTGAGGCCCAGAGACTTG	

**Table 2:** Effect of royal jelly on serum biochemical parameters in different experimental groups of rats

	Control	Rhabdo	Rhabdo +RJ100	Rhabdo +RJ200	Rhabdo +RJ400
CPK (U/l)	635±98	1827±143 ^***^	179.8±46^+++^	252.8±43^+++^	327.8±72^+++^
Urea (mg/dl)	45.14±1.14	458.75±28.8^***^	464.83±18.06	366±37.5	306±55.6^+++##^
Creatinine (mg/dl)	0.55±0.02	4.5±0.3^***^	4.16±0.59	3±0.29^+^	2.28±0.49^+++##^
Potassium (mmol/dl)	5.05±0.11	5.55±0.19	9.2±1.02	5±0.05	5.52±0.46
Calcium (mmol/dl)	11±0.11	13±0.19	1.38±1.02	10.26±0.05	10.75±0.46

All data were expressed as mean ± SEM (n=8 in each group). ***P<0.001 compared to the control group. ++*P*<0.01, +++*P*<0.01 compared to the rhabdomyolysis group. ##*P*<0.01, ###*P*<0.01 compared to Rhabdo+RJ100 group.

Rhabdo: Rhabdomyolysis; RJ: Royal jelly; CPK: Creatine phosphokinase

The values represent mean ± SEM. ***P<0.001 compared to the control group. +*P*<0.05, ++*P*<0.01, and +++ *P*<0.001 significant difference from rhabdomyolysis group, &&*P*<0.01 significant difference from Rhabdo+RJ200 group.

Rhabdo: Rhabdomyolysis; RJ: Royal jelly

The values represent mean ± SEM. ***P<0.001 compared to the control group. +*P*<0.05, ++ *P*<0.01 significant difference from rhabdomyolysis group, #*P*<0.01 significant difference from Rhabdo+RJ100 group.

Rhabdo: Rhabdomyolysis; RJ: Royal jelly; NGAL: Neutrophil gelatinase-associated lipocalin

Values are the mean ± SEM. The data were analyzed using one-way ANOVA and Tukey post hoc test. A significant difference was considered at *P*<0.05. **P<0.01, ***P<0.001 significant difference from control group, +*P*<0.05, ++*P*<0.01 significant difference from rhabdomyolysis group, #*P*<0.05, ##*P*<0.01 significant difference from Rhabdo+RJ100 group, &P<0.05 significant difference from Rhabdo+RJ200 group.

Rhabdo: Rhabdomyolysis; RJ: Royal jelly; IL-1β: interleukin-1β; TNF-α: tumor necrosis factor-α.

The morphology of the kidneys in control animals was normal

Myoglobin casts (thick arrow), hyaline casts (thin arrow), and tubular necrosis were all observed in the Rhabdo group. Treatment with RJ caused mild tubular, glomerular, and interstitial changes in Rhabdo rats (hematoxylin and eosin, ×400). (B) Values represent mean ± SEM. ***P<0.001 compared to the control group. +++*P*<0.001 compared to Rhabdo group.

Rhabdo: Rhabdomyolysis; RJ: Royal jelly

Values are the mean ± SEM. The data were analyzed using one-way ANOVA and Tukey post hoc test. A significant difference was considered at P<0.05. +*P*<0.05, ++ *P*<0.01, +++ *P*<0.001 significant difference from rhabdomyolysis group, &&*P*<0.01 significant difference from Rhabdo+RJ200 group.

Rhabdo: Rhabdomyolysis; RJ: Royal jelly

## Conclusion

Our findings indicated that RJ can mitigate renal damage due to rhabdomyolysis in a dose-dependent manner, likely because of its x anti-oxidant and anti-inflammatory capacities. More studies should be conducted to clarify the mechanisms behind RJ’s beneficial effects on kidney injury related to rhabdomyolysis.

## Declaration

We have not used any AI tools to prepare this manuscript.
